# Therapeutic angiogenesis induced by human umbilical cord tissue-derived mesenchymal stromal cells in a murine model of hindlimb ischemia

**DOI:** 10.1186/s13287-016-0410-4

**Published:** 2016-09-29

**Authors:** Ana Rita S. Pereira, Teresa F. Mendes, Augusto Ministro, Mariana Teixeira, Mariana Filipe, Jorge M. Santos, Rita N. Bárcia, J. Goyri-O’Neill, Fausto Pinto, Pedro E. Cruz, Helder J. Cruz, Susana Constantino Rosa Santos

**Affiliations:** 1 Centro Cardiovascular da Universidade de Lisboa, Av. Prof Egas Moniz 1649-028 Lisbon, Portugal; 2 Centro Hospitalar Lisboa Norte, Av. Prof. Egas Moniz 1649-035 Lisbon, Portugal; 3 ECBio, Investigação e Desenvolvimento em Biotecnologia S.A., R. Henrique Paiva Couceiro, 27, 2700-4511 Amadora, Portugal; 4 Nova Medical School/Faculdade de Ciências Médicas, Universidade Nova de Lisboa, 1169-056 Lisbon, Portugal; 5 Faculdade de Medicina da Universidade de Lisboa, Av. Prof Egas Moniz 1649-028 Lisbon, Portugal

**Keywords:** UCX®, Mesenchymal stem cells, Angiogenesis, Arteriogenesis, Critical limb ischemia, Endothelial cells, Hindlimb ischemia

## Abstract

**Background:**

Mesenchymal stem cells derived from human umbilical cord tissue, termed UCX®, have the potential to promote a full range of events leading to tissue regeneration and homeostasis. The main goal of this work was to investigate UCX® action in experimentally induced hindlimb ischemia (HLI).

**Methods:**

UCX®, obtained by using a proprietary technology developed by ECBio (Amadora, Portugal), were delivered via intramuscular injection to C57BL/6 females after unilateral HLI induction. Perfusion recovery, capillary and collateral density increase were evaluated by laser doppler, CD31 immunohistochemistry and diaphonisation, respectively. The activation state of endothelial cells (ECs) was analysed after EC isolation by laser capture microdissection microscopy followed by RNA extraction, cDNA synthesis and quantitative RT-PCR analysis. The UCX®-conditioned medium was analysed on Gallios flow cytometer. The capacity of UCX® in promoting tubulogenesis and EC migration was assessed by matrigel tubule formation and wound-healing assay, respectively.

**Results:**

We demonstrated that UCX® enhance angiogenesis in vitro via a paracrine effect. Importantly, after HLI induction, UCX® improve blood perfusion by stimulating angiogenesis and arteriogenesis. This is achieved through a new mechanism in which durable and simultaneous upregulation of transforming growth factor β2, angiopoietin 2, fibroblast growth factor 2, and hepatocyte growth factor, in endothelial cells is induced by UCX®.

**Conclusions:**

In conclusion, our data demonstrate that UCX® improve the angiogenic potency of endothelial cells in the murine ischemic limb suggesting the potential of UCX® as a new therapeutic tool for critical limb ischemia.

## Background

Critical limb ischemia (CLI) is a severe form of peripheral artery disease (PAD) in which patients with occlusive arterial disease of the legs experience chronic ischemic rest pain, ulcer, or gangrene [1]. This syndrome is associated with severe prognosis, with 1-year mortality exceeding 25 % and about 30–50 % major limb amputation at 1 year from diagnosis [1]. The limitations of surgical/endovascular revascularization, due the distribution and diffuseness of arterial occlusions, are well recognized and amputation, despite its associated morbidity and mortality rates, is often recommended [2]. The goal of limb salvage has stimulated research into alternative methods, including therapeutic angiogenesis which can be achieved either by local administration of pro-angiogenic growth factors and gene- or cell-based therapies [3].

Mesenchymal stem cells (MSCs) hold great promise as a therapy for PAD, mainly due to their paracrine activity and immunosuppressive capacity [4]. MSCs are known to home specifically to hypoxic tissues following injury [5] where they potentiate vascular growth through the release of pro-angiogenic factors [6]. It was previously reported that autologous, allogeneic and xenogeneic MSC administration induced therapeutic angiogenesis in animal models of hindlimb ischemia (HLI) [7]. In a meta-analysis of cell therapy for PAD, it was found that autologous bone marrow MSCs delivery led to improved indices of ischemia and pain-free walking [8]. Although first harvested from the bone marrow, MSCs have since been identified in many other tissues, namely adipose tissue [9] and umbilical cord tissue [10]. UCX® in particular are MSCs obtained from the human umbilical cord Wharton’s jelly that are isolated, expanded and cryopreserved according to a patented method (PCT/IB2008/054067; WO 2009044379) and produced according to advanced therapy medicinal product (ATMP) guidelines [11]. UCX® fulfil the MSC criteria as defined by the International Society for Cellular Therapy (ISCT) [12]. UCX® cells are advantageous in comparison to other sources due to the absence of invasiveness in their collection process, their faster self-renewal, higher cell yield and being more potent modulators of the immune system than bone marrow MSCs, making them more attractive for allogeneic cellular therapies.

Recently, it was demonstrated that UCX® paracrine activity represses T cell activation and promotes the expansion of regulatory T cells (Tregs) better than bone marrow MSCs [13]. Furthermore, by using an acute arthritis in vivo model it was found that UCX® can reduce paw edema more efficiently than bone marrow MSCs. The use of a chronic arthritis model showed that UCX® induce faster remission of local and systemic arthritic manifestations [13]. The UCX® tissue regeneration capacity suggested for rheumatoid arthritis was later corroborated for myocardial infarction. By using a mouse model of myocardial infarction, it was shown that UCX® preserve cardiac function and attenuate adverse tissue remodelling after intramyocardial transplantation [14]. Interestingly, it was demonstrated that this cardioprotective effect was exerted through paracrine mechanisms involving angiogenesis promotion [14]. Furthermore, in vitro studies, performed with conditioned medium (CM) produced by UCX® grown in classical two-dimensional monolayer cultures, have demonstrated the potential to induce keratinocyte migration in the early stages of wound healing [15]. Moreover, UCX® were able to attract bone marrow MSCs in vivo with potential to promote the formation of granulation tissue, contraction by myofibroblasts, angiogenesis, vasculogenesis and epithelization [15]. These results strongly suggest that UCX® have the potential to promote a full range of events leading to tissue regeneration and homeostasis. Interestingly, it was shown recently that the later proliferative and remodelling stages of wound healing promoted by the CM of the two-dimensional monolayer cultures were improved by using three-dimensional culture-derived CM [16]. An enhanced secretion of healing-inducing paracrine factors by UCX® including the extracellular cellular matrix metalloproteinase-2 (MMP-2) and matrix metalloproteinase-9 (MMP-9), collagen I, fibronectin, laminin, collagen IV and angiogenic factors such as vascular endothelial growth factor A (VEGF-A), granulocyte-colony stimulating factor (G-CSF), transforming growth factor β1 (TGF-β1), fibroblast growth factor 2 (FGF-2), hepatocyte growth factor (HGF) and interleukin 6 (IL-6) was observed in the CM of three-dimensional cultures based on self-assembled spheroids [16].

In this work, our data corroborate that UCX® enhance angiogenesis in vitro and in vivo, by modulating endothelial cells. In vitro, we found that UCX® promote tubule formation and endothelial cell migration. Importantly, our results have demonstrated that UCX® in the setting of experimentally induced unilateral hindlimb ischemia (HLI) stimulate angiogenesis and collateral vessel development and thereby improve blood perfusion in the ischemic limb demonstrating the potential of UCX® as new therapeutic tool for CLI. Furthermore, and to the best of our knowledge, this is the first study in an experimental model of hindlimb ischemia showing that, in vivo and in a sustained way human MSCs upregulate the endothelial gene expression of several pro-angiogenic players (such as transforming growth factor β2 (*Tgfβ2*), angiopoietin 2 (*Ang-2*), *Fgf-2*, *Hgf*), shedding light on the potential mechanisms of action of this particular ATMP.

## Methods

### Ethics and regulations

All animal procedures were performed according to Directive 2010/63/EU. The procedures were approved by the institutional Animal Welfare Body and licensed by DGAV, the Portuguese competent authority for animal protection (license number 023861/2013).

### Cell culture

UCX® were cultured in static monolayers in α-MEM with 1 g/L glucose and 2 mM glutamine (Sigma-Aldrich, St. Louis, MO, USA), hereafter designated basal medium (BM), supplemented with 20 % foetal bovine serum (FBS; Gibco®, Madrid, Spain), in a humidified incubator at 37 °C and 7 % CO_2_.

Human umbilical vein endothelial cells (HUVECs) (Sciencell, Carlsbad, CA, USA) were cultured in M199 media (Sigma-Aldrich, St. Louis, MO, USA), hereafter designated endothelial basal medium (EBM) supplemented with 10 % FBS (Gibco™, Thermo Fisher Scientific, Waltham, MA, USA), 50 μg/ml endothelial cell growth supplement (ECGS) (Sigma-Aldrich), 100 μg/ml heparin (Sigma-Aldrich) and 1 % penicillin-streptomycin (10,000 U/mL, Sigma-Aldrich), hereafter designated endothelial growth medium (EGM). Cells were grown in flasks coated with 0.2 % gelatin (Sigma-Aldrich) until 70 % confluence and used up to passage 6.

### Preparation of conditioned media

UCX® were seeded in BM supplemented with 5 % FBS and grown until 90 % confluence. After washing cells with phosphate-buffered saline (PBS), BM (without serum) was added for 24 hours. Thereafter, BM was replaced and conditioned for 48 hours at 37 °C and 7 % CO_2_ after which it was collected, centrifuged at 300 *g* for 10 minutes to remove cell debris, filtered (0.22 μm pore size, Merck Millipore, Billerica, MA, USA), and concentrated using 5 kDa cut-off spin concentrators (Agilent Technologies, Santa Clara, CA, USA) as per manufacturer’s recommendations. Control conditioned medium samples underwent the same procedure as described above, but in the absence of cells.

### Quantification of secreted factors

The quantification of HGF, VEGF-A, TGF-β1, IL-8, platelet-derived growth factor-AA (PDGF-AA) and FGF-2 in the conditioned medium was performed using a commercially available kit (FlowCytomix™; eBioscience, San Diego, CA, USA) according to the manufacturer’s instructions. Samples were acquired on a Gallios flow cytometer (Beckman Coulter, Brea, CA, USA) and the results obtained using FlowCytomix Pro 3.0 Software (eBioscience).

### Tubule formation assay

The tubule formation assay was performed using the thick gel method of preparation. Briefly, after thawing overnight, the matrigel (Corning, Corning, NY, USA) was plated into a pre-cooled 48-well plate (Nunc™, Thermo Fisher Scientific) (180 μl per well) using a chilled pipet tip. After polymerization of matrigel at 37 °C, 5 % CO_2_, for 45 minutes, HUVECs were inoculated at a density of 4.5 × 10^4^ cells/cm^2^ on top of the matrigel in 350 μl of EBM. Cells were then incubated for 1 hour at 37 °C, 5 % CO_2_. 1 × 10^6^ UCX® were resuspended in 350 μl BM and loaded on a 1-μm insert (Brand, Wertheim, Germany) and carefully placed in a 48-well plate and incubated for 16 hours at 37 °C, 5 % CO_2_. Controls with no UCX® included HUVECs in (i) EBM, (ii) EGM and (iii) EBM supplemented with FGF-2 (50 ng/ml). After incubation, inserts were removed and all wells were photographed (×10 amplification) using a Nikon Eclipse Ti-U inverted microscope with a conjugated Nikon DS-Qi1Mc camera (Nikon, Tokyo, Japan). Tubule formation was quantified on four random fields per replicate, using the Angiogenesis analyser from Image J.

### Wound-healing assay

HUVECs were plated to confluence in a 24-well plate (previously coated with gelatine 0.2 %) with EBM supplemented with 5 % FBS. After culturing overnight, wounds were created in the monolayer by scrapping the plate with a sterile pipette tip. After that, 1 × 10^6^ UCX®, resuspended in EBM supplemented with 5 % FBS and loaded on a 1-μm insert (Brand, Wertheim, Germany), were carefully placed in a 24-well plate and incubated for 9 hours at 37 °C, 5 % CO_2_. In controls, the same procedure was performed in the absence of UCX®. Photographs were taken in Primovert (Carl Zeiss Microscopy, Jena, Germany) on a × 4 magnification and three independent wound areas were measured with ImageJ immediately after wounding and 9 hours later.

### Mice

Twenty-two-week-old C57BL/6 female mice, purchased from Charles River Laboratories, Barcelona, Spain, were used in all experiments. The animals were anaesthetised with a ketamine-medetomidine cocktail (75 mg/kg BW and 1 mg/kg BW, respectively) intraperitoneally for the surgical procedure as well as for the other analysis procedures. The anaesthesia was partially reverted with atipamezole (5 mg/kg BW). Postoperatively, analgesia was performed (buprenorphine 100 μl/15–30 g BW every 8–12 h) and the animals were closely monitored.

### HLI model

A surgical procedure was performed to induce unilateral HLI in the mice. Briefly, an incision in the skin overlying the thigh of the right hindlimb of each mouse was made and the distal external iliac artery and the femoral artery and veins were ligated and excised. The vein was ligated both to increase the severity of the ischemia as well as to increase the technical reproducibility of the model, as isolation of the femoral artery alone often results in tearing of the vein resulting in haemorrhage.

### UCX® administration

A dose of 2 × 10^5^ UCX® was injected intramuscularly, 5 hours post-ischemia induction on the gastrocnemius muscle (right hindlimb) in a volume of 50 μl per animal, divided into two injections of 25 μl. As a control, UCX® vehicle (PBS) was administered at the same conditions.

### Laser doppler perfusion imaging

The laser doppler perfusion imager (MoorLDI V6.0, Moor Instruments Ltd, Axminster, UK) was used to assess limb perfusion. Hair was removed 1 day before laser doppler analysis using an electrical shaver followed by depilatory cream. Blood flow was measured both in the ischemic leg and the contralateral one, before HLI induction (PRE-HLI), immediately post-HLI (POST-HLI), and at day 7, 14 and 21 post-HLI (d7 POST-HLI, d14 POST-HLI and d21 POST-HLI, respectively). Colour-coded images of tissue perfusion were recorded and poor or no perfusion was displayed as dark blue, and the highest perfusion level was displayed as red. Mean flux values were calculated using the MoorLDI V6.0 image processing software. To account for variables such as temperature and ambient light, blood perfusion is expressed as the ratio of ischemic to non-ischemic limb. The mice were placed on a 37 °C heating pad to reduce heat loss during measurements.

### Immunohistochemistry and capillary density analysis

Mice were sacrificed at day 90 post-HLI (to assure capillary stabilization after the ischemic injury). The gastrocnemius muscles of both legs were harvested, placed in transverse orientation on a small cork disc with the help of 10 % tragacanth, snap frozen in liquid nitrogen-cooled isopentane and stored at -80 °C until sectioned. Seven-micrometer sections were labeled with CD31 monoclonal antibody (Pharmingen, San Diego, CA, USA). After fixation in acetone for 10 minutes, hydrogen peroxidase (0.3 % diluted in methanol) was added for 30 minutes, at room temperature (RT) and followed by two washes in PBS for a total of 10 minutes. Blocking solution (5 % rabbit serum in PBS) was applied for 30 minutes, at RT, and the slides were then incubated for 1 hour at RT with rat monoclonal antibody against mouse CD31 at 1:500, diluted in 1 % bovine serum albumin (BSA) in PBS. After three washes in PBS for a total of 30 minutes, a secondary biotinylated rabbit anti-rat IgG antibody was added at 1:200 in 1 % BSA in PBS and 5 % rabbit serum for 30 minutes, at RT. Washes were performed as before and labeled avidin-conjugated peroxidase complex (Vectastain ABC kit; Vector Laboratories, Burlingame, CA, USA) was used for color development according to the manufacturer’s recommendations for 30 minutes, at RT. After rinsing in PBS (three times for 5 minutes), DAB peroxidase substrate kit (Dako, Glostrup, Denmark) was added for 5 minutes to localize the immune complexes. The sections were counterstained with haematoxylin (Merck, Kenilworth, NJ, USA) for 10 seconds and mounted with entelan (Merck). Omission of the first antibody was used as a negative control. Analysis of tissue samples was conducted using a Leica DM2500 upright brightfield microscope (Leica Microsystems, Wetzlar, Germany). Capillary densities, i.e. number of capillaries per number of myocytes, were measured in two different sections of four distinct anatomic areas of each specimen using the ImageJ software.

### Contrast agent perfusion and diaphonisation

Ninety days post-ischemia induction (to assure vascular stabilization after the ischemic injury), mice were deeply anaesthetised and the torso and limbs were shaved. A medial thoracotomy was performed to expose the heart and a needle (26 gauge), attached to an automatic injector, was introduced in the left ventricle. An incision was performed in the right atrium to allow venous drainage. Mice were primarily perfused with heparinized serum (3000 IU/L) until the blood was completely removed from circulation. A vasodilatation mixture of adenosine (1 mg/L) and papaverine (4 mg/L) was subsequently administered, right before the contrast agent, for 2 minutes. The contrast used was a mixture of barium sulfate (50 %) and gelatin (5 %). This solution was kept warm until the injection time to avoid thickening. Contrast agent was perfused manually until the feet blanched. Right after the injection the mice were transferred to a cold chamber, so that the contrast agent became solidified. All the solutions were injected with a perfusion rate of 0.7 mL/minute. Then, the skin of each mouse was removed from the lower body and diaphonisation was performed by using a modified version of the Spalteholz technique. Briefly, the mice were fixated, decalcified, whitened, washed, dehydrated by freeze substitution and placed into a vacuum pump with Spalteholz solution (benzyl benzoate and methyl salicylate) until transparency was acquired.

### Collateral vessels quantification

Mice were kept in Spalteholz solution during image acquisition, in order to achieve a homogenous density between the tissues and the media, with minimal absorption or reflection of light. Mice entire limbs were photographed in a magnifier with a light source. After acquisition, images were aligned and stitched together using Adobe Photoshop CS6®, and entire limb photographs were obtained. In order to exclude the femoral artery and all venous structures from this quantification, only the collateral vessels were manually segmented by highlighting them using Adobe Photoshop CS6®. We considered as collateral vessels all the vessels with a diameter between 20 and 300 μm. For every mouse, an anatomically determinable region comprising the ligation site was selected and defined as a region of interest (ROI). Collateral vessels density (CVD) was quantified in equivalent ROIs corresponding to 20 % of the total limb area. The CVD was calculated as the ratio between the vascular and the ROI areas.

To exclude variations in the anatomy, perfusion and diaphonization procedures, the CVD value of the non-ischemic limb obtained for each mouse was assumed to correspond to 100 %. According to this assumption, the CVD percentage in the ischemic limb was calculated relatively to the non-ischemic one. The percentage of CVD increase was determined as the difference between the CVD percentage among the ischemic and non-ischemic limbs. Means of CVD percentages and standard deviations were calculated for each experimental group. All density measurements were performed using ImageJ software.

### Laser capture microdissection of capillaries

Mice were sacrificed at day 70 post-HLI. Twelve-micrometer sections of the gastrocnemius muscles were labeled with CD31 monoclonal antibody (Pharmingen). The sections were stored at –80 °C until microdissection. The immunohistochemistry protocol described above was modified to improve RNA preservation by using high salt buffer, 2 M NaCl in PBS 1× (at 4 °C) in all incubation and washing steps [17]. Briefly, slides were placed in acetone, for 5 minutes, air-dried, rehydrated with 2 M NaCl/PBS (4 °C) and incubated, overnight, at 4 °C with rat monoclonal antibody against mouse CD31, at 1:500, in 2 M NaCl/PBS. After two washes in 2 M NaCl/PBS for a total of 6 minutes, a secondary biotinylated rabbit anti-rat IgG antibody was added at 1:200 in 2 M NaCl/PBS and 5 % rabbit serum for 30 minutes, at 4 °C. Washes were performed as before and labeled avidin-conjugated peroxidase complex (Vectastain ABC kit; Vector Laboratories) was used for color development according to the manufacturer’s recommendations for 30 minutes, at 4 °C. After rinsing, DAB peroxidase substrate kit (Vector Laboratories) was added for 5 minutes to localize the immune complexes. Sections were dehydrated in ice-cold 90 % ethanol followed by 100 % ethanol and allowed to dry. Ten thousand capillaries were microdissected using a Zeiss PALM MicroBeam Laser Microdissection System (Carl Zeiss Microscopy) equipped with a pulsed solid-state 355 nm laser. Dissected capillaries were catapulted into a microfuge tube adhesive cap.

### RNA extraction, cDNA synthesis, pre-amplification and RT-PCR

Total RNA from the microdissected capillaries was isolated using an RNeasy Micro Kit (QIAGEN, Hilden, Germany), including DNase treatment to remove potential genomic DNA contamination. For synthesis and preamplification of cDNA, RT^2^ PreAMP cDNA Synthesis Kit (QIAGEN) was used with two rounds of pre-amplification using the following primers: *Fgf-2*_F (5′- ACTCCAGTTGGTATGTGGCACTGA-3′); *Fgf-2*_R (5′-AACAGTATGGCCTTCTGTCCAGGT-3′); *Tgfb-2*_F (5′-GCTTTGGATGCGGCCTATTGCTTT-3′); *Tgfb-2*_R (5′-CTCCAGCACAGAAGTTGGCATTGT-3′); *Ang-2*_F (5′-ATCCAACACCGAGAAGATGGCAGT-3′); *Ang-2*_R (5′-AACTCATTGCCCAGCCAGTACTCT-3′); *Hgf*_F (5′-GCATTCAAGGCCAAGGAGAAGGTT-3′); *Hgf*_R (5′-TCATGCTTGTGAGGGTACTGCGAA-3′); *18s*_F (5′-GCCCTATCAACTTTCGATGGTAGT-3′); *18s*_R (5′- CCGGAATCGAACCCTGATT-3′). RT-PCR was performed according to the manufacturer’s protocol using Power SYBR® Green (Invitrogen, Carlsbad, CA, USA) and an Applied Biosystems (Waltham, MA, USA) 7500 Fast Real-Time PCR for the same targets described above. The housekeeping gene used to normalize was 18S. The RT-PCR program consisted of an initial denaturation step, at 95 °C, for 10 minutes followed by 50 cycles, at 95 °C, for 15 seconds and at 60 °C, for 1 minute. The relative quantification was performed according to the comparative method (2^-ΔΔCt^; Applied Biosystems User Bulletin no. 2P/N 4303859), with the non-ischemic muscle as an internal calibrator. The formula used is 2^-ΔΔCt^ =2^-[ΔCt (sample) - ΔCt (calibrator)]^, where ΔCt (sample) = Ct (sample) –Ct (reference gene). For the internal calibrator ΔΔCt = 0 and 2^0^ = 1. For the remaining samples the value of 2^-ΔΔCt^ indicates the fold change in gene expression relative to the calibrator. The ΔCt value for each sample is the average of triplicates.

### Statistical analysis

For the wound-healing assay, the values assume normal distribution and unequal variances and therefore an independent two-tailed *t* test was performed. For doppler analysis, an independent two-tailed *t* test was also performed at days 7, 14 and 21. The values assume normal distribution. Only at day 7, we cannot assume equal variances. For capillary and collateral analysis, the values also assume normal distribution and equal variances and therefore an independent two-tailed *t* test was also performed. For the master junctions and segment length, one-way independent analysis of variance (ANOVA) was developed as data followed a normal distribution, though equality of variances could not be assumed for the first. Therefore, a Games-Howell corrected post hoc test was used to identify differences between groups. For the meshes area, as normality could not be assumed and so a Kruskal-Wallis non-parametric test was developed followed by its paired analysis to identify differences between groups. The effect size and power was determined by using the G-Power software in all type of analyses. *p* < 0.05 was interpreted to denote statistical significance.

## Results

### UCX® promote tubulogenesis and endothelial cell migration

**Fig. 1:**
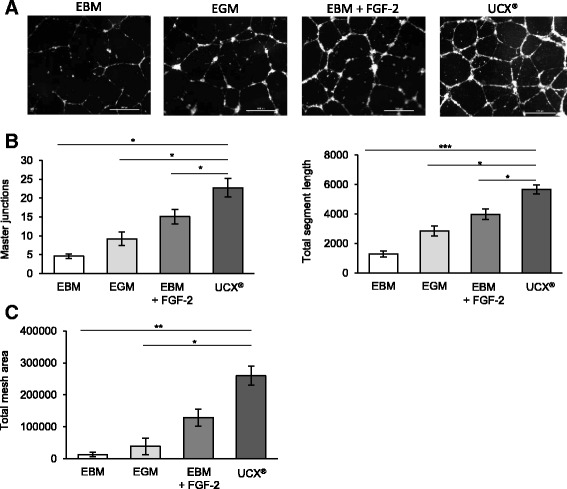
UCX® promote tubulogenesis. Matrigel assay was performed by seeding human umbilical vein endothelial cells (HUVECs) in endothelial basal medium (EBM), endothelial growth medium (EGM), EBM supplemented with fibroblast growth factor 2 (FGF-2) (EBM + FGF-2) or EBM co-cultured with UCX®. (**a**) Representative images from the different experimental conditions are shown. (**b**) Quantitative evaluation demonstrated significantly enhanced master junctions, total mesh area and total segment length in capillary-like structures formed in HUVECs co-cultured with UCX® when compared to the other experimental conditions. For master junctions, following Games-Howell post hoc test the mean differences between UCX® and the other experimental conditions were: EBM (mean dif. =24.27; *p* = 0.01); EGM (mean dif. = 19.67; *p* = 0.03) and EBM + FGF-2 (mean dif. = 13.8; *p* = 0.034). The effect size to basal was 2.08 and power 0.98. For the meshes area, following pairwise comparisons, the equivalent to a post hoc Kruskal-Wallis test, significant differences between UCX® and EBM or UCX® and EGM were observed with KW = 22.66, *p* < 0.01 or KW = 18.77, *p* = 0.01, respectively. For segment length, following Games-Howell post hoc test, UCX® show significant difference when compared to the other groups: EBM (mean dif. = 4920; *p* < 0.001); EGM (mean dif. = 3346; *p* = 0.01) and EBM + FGF-2 (mean dif. = 2223; *p* = 0.016). Effect size to basal was 3.85 and power 1. Scale bar, 500 μm

**Fig. 2:**
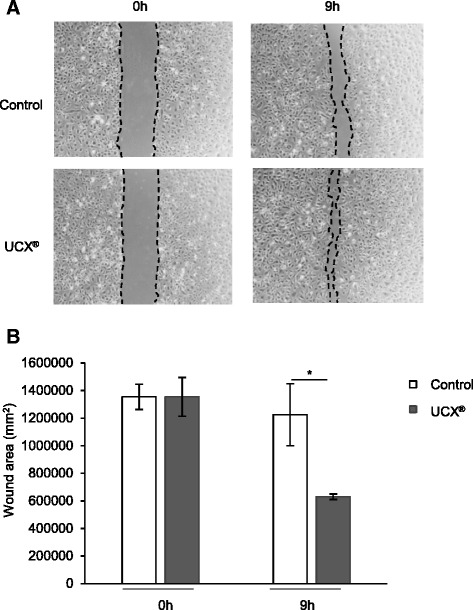
UCX® stimulate endothelial cell migration. Human umbilical vein endothelial cells (HUVECs) were seeded to confluence in endothelial basal medium (EBM) supplemented with 5 % FBS and cultured for 16 hours after wounding. Immediately after wound, HUVECs were co-cultured with UCX® or not (control) for 9 hours. (**a**) Representative images are shown. The *left* and *right panels* concern HUVEC monolayer immediately after or 9 hours post-wounding, respectively. (**b**) The wound area was measured immediately after and 9 hours post-wounding by using ImageJ software (three independent measurements were performed for each experimental group; t (2.03) = 4.66; *p < 0.05); effect size was 3.82 and power 0.93)

We first evaluated the effect of UCX® on the capillary structure formation. A matrigel tubule formation assay was used as an in vitro model. HUVECs were seeded onto matrigel in endothelial basal medium (EBM) and co-cultured with UCX®. As a control HUVECs were seeded in EBM, where we do not expect to visualize capillary-like structures formed by HUVECS. For that reason, two positive controls were added and HUVECs were seeded both in endothelial growth medium (EGM) that contains several pro-angiogenic factors such as VEGF and FGF-2 and in EBM supplemented with FGF-2 (EBM-FGF-2). As shown in Fig. 1a and quantified in Fig. 1b, UCX® induce tubule formation by HUVECs and these structures consistently showed a significant increase in the number of master junctions, total mesh area and total segment length when compared to those from HUVECs cultured in EBM or EGM. With the exception of the total mesh area, all the measured parameters were also significantly different between HUVECs cultured with UCX® in EBM and HUVECs cultured in EBM supplemented with FGF-2. Next, we assessed the migratory capacity of HUVECs seeded in EBM supplemented with 5 % of serum co-cultured with UCX® without cell to cell contact. With this objective, a wound-healing assay was performed and as a control HUVECs were seeded in the same culture conditions but in the absence of UCX®. As shown in Fig. 2a and b, the wound area decreased more rapidly in the presence of UCX® than in the control condition, suggesting that UCX® promote the migration of HUVECs via a paracrine secretion. Taken together, these results clearly show that, in vitro, UCX® induce angiogenic processes in endothelial cells.

### UCX® secrete pro-angiogenic factors

Since it was already demonstrated that the beneficial effects promoted by UCX® result from a paracrine secretion, we decided to evaluate the secretion of six representative trophic factors critical for angiogenesis in the conditioned medium of an UCX® monolayer seeded in basal medium (BM) for 48 hours. As a control, the same trophic factors were measured in BM that had not been in contact with cells. The expression of HGF, VEGF-A, TGF-β1, interleukin 8 (IL-8), platelet-derived growth factor-AA (PDGF-AA) and FGF-2 were analysed. Our results show a significant increase of HGF, TGF-β1, IL-8 and PDGF-AA in the conditioned medium of UCX® when compared to the control (Table 1), demonstrating that UCX® secrete pro-angiogenic factors to the extracellular medium.

**Table 1:** Pro-angiogenic factors present in the conditioned medium from UCX® or basal medium

	UCX® CM (pg/ml) Mean±SEM	Basal CM (pg/ml) Mean±SEM	t-test
HGF	2,64 ± 0,718	0,001 ± 0	0,0144
VEGF-A	2,025 ± 1,043	0,001 ± 0	0,1099
TGF-β1	0,983 ± 0,122	0,001 ± 0	0,0005
IL-8	0,765 ± 0,069	0,001 ± 0	0,0001
PDGF-AA	0,123 ± 0,045	0,001 ± 0	0,0412
FGF-2	0,0317 ± 0,009	0,02 ± 0	0,2874

*CM conditioned medium*

### UCX® increase perfusion recovery after HLI

**Fig. 3:**
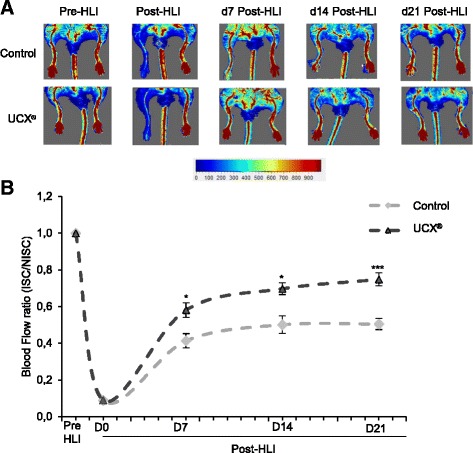
UCX® increase perfusion recovery. UCX® or their vehicle (as a control) were administered in the ischemic gastrocnemius muscle 5 hours after HLI induction. (**a**) Representative laser doppler flow images before (PRE-HLI), immediately after (d0 POST-HLI) and at 7, 14 and 21 days post-HLI induction (d7 POST-HLI, d14 POST-HLI, d21 POST-HLI). (**b**) Quantitative evaluation of blood flow expressed as a ratio of ISC to NISC limb demonstrated significantly enhanced limb blood perfusion in UCX®-treated mice at 7, 14 and 21 days post-HLI. (n = 16 for each experimental group; D7: t (22.69) = 4.26; ***p < 0.001; effect size was 1.51 and power 0.98; D14: t (30) = 4.7; ***p < 0.001; effect size was 1.66 and power 0.99; D21: t (30) = 7.22; ***p < 0.001; effect size was 2.56 and power 0.99). *HLI* hindlimb ischemia, *ISC* ischemic, *NISC* non-ischemic

In order to evaluate the therapeutic potential of UCX® in a HLI mouse model, the perfusion recovery was assessed over time after ischemia induction and UCX® administration. Five hours after surgical induction of unilateral HLI, UCX® or their vehicle (as a control) were administered in the ischemic muscle and perfusion was measured over time. As shown in Fig. 3a and quantified in Fig. 3b, a dramatic reduction in blood flow was observed in the ischemic limb immediately after surgery, in comparison to the contralateral limb, followed by a gradual normalization of ischemic blood flow over time. Importantly, UCX® markedly improved blood flow recovery at 7, 14 or 21 days post-HLI, comparing with control mice, demonstrating that UCX® administration contributes toward the functional recovery of ischemic tissues.

### UCX® increase capillary and collateral vessel density after HLI

**Fig. 4:**
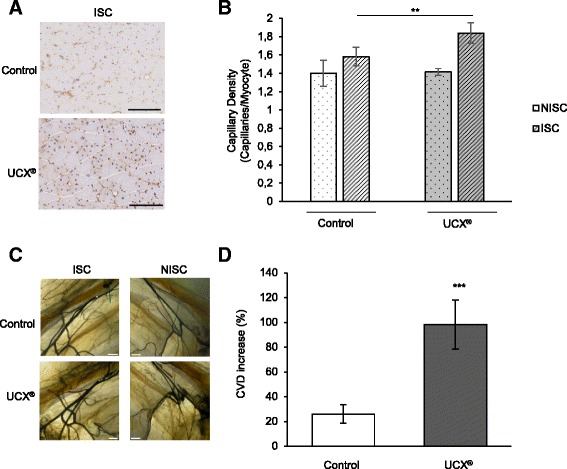
UCX® increase capillary and collateral densities. UCX® or their vehicle (as a control) were administered in the ischemic gastrocnemius muscle 5 hours after HLI induction. (**a**) Representative sections from control and UCX®-treated ischemic gastrocnemius muscles at 90 days post-HLI. Capillaries and myocytes were identified by CD31 immunohistochemistry and haematoxylin, respectively. Scale bar, 125 μm. (**b**) Quantitative analysis, at 90 days post-HLI, revealed increased capillary density (capillaries/myocyte) in UCX®-treated ischemic gastrocnemius muscles compared to control ischemic ones. (n = 3 and n = 7 for control and UCX®, respectively; t (8) = 4.44; **p = 0.0057; effect size was 3.064 and power 0.97). (**c**) Illustrative images of selected regions of interest (ROI) for control and UCX®-treated mice. ISC and NISC limbs are shown. Scale bar, 1 mm (**d**) Data are represented as the percentage of collateral vessel density (CVD) increase of the ISC limb relatively to the NISC one. At 90 days post-HLI, UCX®-treated mice presented significantly higher CVD increase (%) when compared to control mice (n = 5 for each experimental group; t (8) = 7.63; ***P = 0.000062; effect size was 4.82 and power 0.99). *ISC* ischemic, *NISC* non-ischemic

As blood flow recovery depends on both angiogenesis and arteriogenesis we examined whether UCX® would affect capillary and collateral vessel densities in hindlimb muscles. Five hours after surgical induction of unilateral HLI, UCX® or their vehicle (as a control) were administered in the ischemic muscle and mice were sacrificed 90 days post-HLI induction. Capillary density was assessed through quantification of CD31-positive capillaries on histological sections of gastrocnemius muscle. As expected, the capillary density was greater in the ischemic versus the non-ischemic hindlimb in both experimental groups (Fig. 4b). Notably, the level of capillary density in the ischemic hindlimb treated with UCX® was significantly higher than the one observed in the control ischemic hindlimb, as shown in Fig. 4a and quantified in Fig. 4b. In order to evaluate the collateral vessel density (CVD), mice were diaphonised and an equivalent region of interest (ROI), corresponding to 20 % of the limb area, was selected for CVD quantification (Fig. 4c). A significantly higher CVD increase was observed in UCX®-treated mice when compared to the control (Fig. 4d). Taken together, our results show that UCX® significantly augment capillary density and CVD increase after ischemic injury.

### UCX® upregulate the endothelial *Tgf-β2, Ang-2, Fgf-2* and *Hgf* expression in response to HLI

**Fig. 5:**
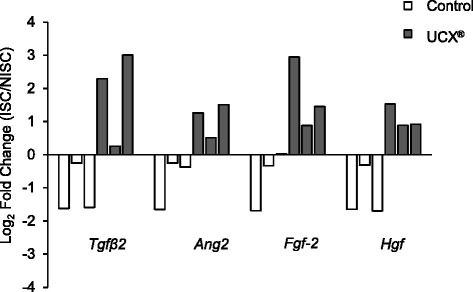
UCX® upregulate the expression of *Tgf-β2, Ang-2, Fgf-2* and *Hgf* in endothelial cells isolated from ischemic gastrocnemius muscles. UCX® or their vehicle (as a control) were administered in the ischemic gastrocnemius muscle 5 hours after HLI induction. At 70 days post-HLI, the expression of pro-angiogenic factors and their receptors was evaluated by qRT‐PCR exclusively on endothelial cells isolated from gastrocnemius muscles. Each bar represents the relative gene expression in one animal*. White* and *grey bars* represent control and UCX®-treated mice, respectively. Values were normalized to 18S to obtain relative expression levels. Results expressed as log2 fold changes between ISC and NISC samples demonstrated relative abundance of the transcripts in UCX®-treated mice; in contrast, a downregulation is observed in control mice*. ISC* ischemic, *NISC* non-ischemic

To elucidate the mechanism of action of UCX® in our HLI model we investigated if UCX® could change the endothelial gene expression. For this purpose, 5 hours after surgical induction of unilateral HLI, UCX® or their vehicle (as a control) were administered in the ischemic muscle and mice were sacrificed at day 70 post-HLI induction. The gastrocnemius muscle sections were stained for CD31 and visualized using a Laser Capture Microdissection microscope. CD31-positive cells were dissected, isolated and analysed by quantitative RT-PCR for the expression of several pro-angiogenic factors*.* Our results show that transcripts for *Tgf-β2, Ang-2, Fgf-2* and *Hgf* were clearly upregulated in endothelial cells isolated from muscle of the ischemic limb, comparing with those found in endothelial cells from the contralateral limb. This is observed exclusively in mice treated with UCX®. Control mice show the opposite trend, downregulating the expression of the angiogenic genes in endothelium from the ischemic limb, when compared with the contralateral limb (Fig. 5). These results suggest that the mechanism of action of UCX® in the HLI mouse model involves the upregulation of several angiogenic genes in the endothelial cells present in the ischemic muscle.

## Discussion

CLI is a manifestation of peripheral arterial disease that describes patients with chronic ischemic rest pain, ulcers or gangrene [1]. Available therapies are limited and patients may require amputation. The goal of limb salvage has stimulated research into alternative methods, including therapeutic angiogenesis. Stem cell therapy holds great potential for therapeutic angiogenesis, but its clinical translation has been slow due to (i) lengthy procedures performed ex vivo in which stem cells are manipulated and often lose viability, differentiate or change their characteristics, and (ii) the high costs involved. In addition, the clinical use of bone marrow MSCs has demonstrated some caveats, mainly due to low yields which further decrease with donor’s age and medical condition, leading to variable and/or limited cell doses.

Among the other possible sources for MSCs, there is the umbilical cord tissue [10], from which UCX® are derived. UCX® have already demonstrated the potential to lead to tissue regeneration and homeostasis, promoting faster remission of local and systemic manifestations of inflammatory arthritis [13]; preserving cardiac function after intramyocardial transplantation in a myocardial infarction murine model [14]; and accelerating wound healing [15]. Those studies strongly suggest that UCX® act in different cell types through paracrine mechanisms. In addition, it was found that conditioned medium of UCX® cultures induces fibroblast and keratinocyte migration and UCX® are chemotactic to CD34^-^/CD45^-^ bone marrow MSCs [15]. Further, it was also shown that in vitro UCX®-conditioned medium induces angiogenesis by promoting the formation of capillary-like structures by HUVECs [13]. Herein, we corroborated those findings showing that UCX® promotes tubulogenesis and endothelial cell migration. We observed that UCX® when co-cultured with HUVECs induce tubule formation by HUVECs to a higher extent than the known pro-angiogenic factor FGF-2, and also promote migration. As already demonstrated for other cell types, our data strongly suggest that UCX® act in endothelial cells through paracrine mechanisms since a significant increase of pro-angiogenic factors such as HGF, TGF-β1, IL-8 and PDGF-AA was observed in the conditioned medium of UCX® when compared to the control.

In order to investigate if UCX® could promote therapeutic angiogenesis in a HLI context, we developed an experimental model of unilateral HLI and two important parameters were assessed: angiogenesis and arteriogenesis. Our data indicate that UCX® administration significantly induces blood perfusion recovery in the limb after HLI induction. Accordingly, a significantly higher capillary density and CVD increase was found in the ischemic muscles after UCX® treatment. These evaluations were performed at day 90 post-HLI suggesting that the effect induced by UCX® in angiogenesis and arteriogenesis is maintained over time. Besides, the non-ischemic muscles are unaffected by UCX® since capillary density and CVD are similar in non-ischemic muscles both from UCX®-treated and control mice, indicating that UCX® might have a local action. These data suggest that UCX® could secrete cytokines/chemokines and paracrinally interfere with the ischemic microenvironment contributing to a therapeutic angiogenesis. More importantly, a new mechanism of action of UCX® is demonstrated for the first time: UCX® modulate the expression of pro-angiogenic players in endothelial cells. We found that 70 days post-HLI and UCX® treatment, endothelial cells isolated from UCX®-treated microenvironment present an upregulation in their expression levels for *Tgfβ2, Ang-2, Fgf-2* and *Hgf* when isolated from the ischemic gastrocnemius muscle in comparison with the contralateral one. This effect is UCX®-specific as the opposite is verified in control mice. According to these findings, we show that UCX® simultaneously upregulate the expression of several angiogenic factors in endothelial cells and by this mechanism we may hypothesize that UCX® improve their endogenous angiogenic potency in an ischemic context. This could be very relevant when compared to other strategies where only a single angiogenic factor is administered, especially if we understand angiogenesis as a complex process that involves multiple cytokines. It is also very interesting to note that the same factors modulated by UCX® have been extensively studied in therapeutic angiogenesis. Specifically, in gene and protein therapy, FGF-2 was used in a rabbit model of HLI [18], where it was shown to promote the development of collateral vessels. The efficacy of FGF-2 was also evaluated in clinical trials such as FIRST (FGF Initiating Revascularization Trial) [19] and TRAFFIC (Therapeutic Angiogenesis with Recombinant FGF-2 for Intermittent Claudication) [20]; however, it did not show a sustained success [21]. Concerning HGF, it was also used in clinical trials through gene transfer to treat CLI, where its safety was shown [22]. More rigorous controlled trials (randomised and placebo-controlled studies with larger numbers of patients) are currently ongoing. Given the fact that CLI is a common complication of diabetes mellitus, future studies should assess the UCX® efficacy in a diabetic experimental model since diabetes may adversely affect EPC function and thus limit therapeutic efficacy. Recent data have further shown that the secreted proangiogenic protein osteopontin (OPN), significantly downregulated in diabetic EPCs, could increase the secretion of angiogenic proteins from EPCs increasing their therapeutic efficacy [23]. Importantly, these findings show that the function of EPCs could be modulated and raise interesting questions for future investigation, such as, (a) which molecules could eventually improve the therapeutic efficacy of UCX® in an ischemic microenvironment; (b) could different ischemic microenvironments modulate differently the function of UCX®; if so, by which mechanisms; and (c) should UCX® be modulated by the administration of selected molecules according to the pathologies associated with CLI.

## Conclusions

In conclusion, here we propose a model of enhanced and sustained angiogenesis induction by using UCX® as a promising therapeutic approach for CLI. UCX® significantly induce blood perfusion, capillary density and collateral development. This could be at least in part achieved by a new mechanism since we show that UCX® upregulate the simultaneous expression of several pro-angiogenic factors in endothelial cells that could improve their angiogenic potency.

## Abbreviations

ATMP: advanced therapy medicinal product
Ang-2: angiopoietin 2
BM: basal medium
CLI: critical limb ischemia
CM: conditioned medium
CVD: collateral vessels density
EBM: endothelial basal medium
EGM: endothelial growth medium
ECs: endothelial cells
FGF-2: fibroblast growth factor 2
G-CSF: granulocyte-colony stimulating factor
HGF: hepatocyte growth factor
HLI: hindlimb ischemia
HUVECs: human umbilical vein endothelial cells
IL-6: interleukin 6
IL-8: interleukin 8
MMP-2: matrix metalloproteinase-2
MMP-9: matrix metalloproteinase-9
MSCs: mesenchymal stem cells
PAD: peripheral artery disease
PDGF-AA: platelet-derived growth factor-AA
POST-HLI: post-HLI
PRE-HLI: before HLI
ROI: region of interest
RT: room temperature
TGF-β1: transforming growth factor β1
Tgfβ2: transforming growth factor β2
VEGF-A: vascular endothelial growth factor A
